# Multimodal biomarkers in idiopathic sudden sensorineural hearing loss: integrating immune, vascular, and metabolic perspectives for precision medicine

**DOI:** 10.3389/fimmu.2026.1880059

**Published:** 2026-07-10

**Authors:** Qian Li, Hang Li, Haiyuan Zhao

**Affiliations:** 1 Department of Clinical Laboratory, Kaifeng Central Hospital, Kaifeng, China; 2 Department of Otorhinolaryngology, Kaifeng Central Hospital, Kaifeng, China

**Keywords:** biomarkers, idiopathic sudden sensorineural hearing loss, laboratory diagnosis, metabolomics, sensorineural hearing loss

## Abstract

Idiopathic Sudden Sensorineural Hearing Loss (ISSNHL) is a common otological emergency driven by intertwined microcirculatory disorders, immune-inflammatory activation and metabolic dysregulation.This narrative overview collates representative published evidence to summarize research advances on hematological, immunological and metabolomic biomarkers, with a focus on their investigational—not yet clinically validated— diagnostic and prognostic potential. Key observational findings from existing cohorts are outlined as follows: (i) inflammatory and coagulation markers display promising discriminatory performance in single-center exploratory analyses, yet consistent cut-off values across populations remain absent; (ii) multi-indicator combined nomograms yield favorable predictive metrics within isolated research groups, though external multi-center validation is still required; (iii) exosomal molecules, complement C3, and other novel metabolic markers provide new analytical angles to interpret disease mechanisms and guide tentative individualized intervention research. Despite these preliminary observational results, widespread clinical translation is hindered by limited sample scales, non-uniform detection protocols and confounding comorbidities. Future multi-omics combined modeling aided by artificial intelligence may only serve as auxiliary stratification tools after rigorous prospective verification, rather than supporting definitive clinical classification at the current stage.This narrative collation merely offers preliminary analytical references for auxiliary clinical assessment and personalized research design of ISSNHL, without claiming validated diagnostic utility for routine practice.

## 1 Introduction

Idiopathic Sudden Sensorineural Hearing Loss (ISSNHL), the most common form of Sudden Sensorineural Hearing Loss (SSNHL), refers to a decrease in hearing of at least 30 decibels over three consecutive test frequencies within 72 hours (1). It is most common in people aged 30–50 years, can affect all age groups, is predominantly unilateral, with bilateral cases accounting for 2%-3% (mostly secondary, but can also occur simultaneously, with no significant difference in sex or side distribution) (2). In 70-90% of SSNHL cases, it is idiopathic, i.e., Idiopathic Sudden Sensorineural Hearing Loss (ISSNHL) (3, 4). As a common otological emergency in otorhinolaryngology, the annual incidence is 11–77 cases per 100,000 people. When the average pure-tone interaural difference is ≥40 dB, ISSNHL is the most common cause of severe unilateral hearing loss (5). To date, the mainstream hypotheses regarding the etiology and pathogenesis of ISSNHL suggest that it is related to multiple factors, including: microcirculatory disorders and vascular lesions, autoimmune reactions, viral infections, metabolic diseases, and other factors. However, no single theory can fully explain all cases (6–11). For example, vascular ischemia is considered one of the most likely causes of acute labyrinthine ischemia (12); viral infections such as varicella-zoster virus, cytomegalovirus, Epstein-Barr virus, and even SARS-CoV-2 have also been reported to be associated with SSNHL (13–16); in addition, immune factors are also involved, and autoimmune inner ear disease is considered one of the causes of SSNHL, although its proportion may be less than 1% (3, 17).Terminology note: Throughout this narrative overview, ‘ISSNHL’ is used when referring specifically to the idiopathic subset of sudden sensorineural hearing loss. Several cited studies enrolled patients under the broader ‘SSNHL’ designation (which includes both idiopathic and non-idiopathic cases). Given that 70%–90% of SSNHL cases are idiopathic, findings from such studies are discussed in the context of ISSNHL, with the original enrolment criteria noted where relevant. Readers should consult the original articles for exact inclusion and exclusion criteria. In terms of diagnosis, according to current clinical practice guidelines, the diagnostic workup for ISSNHL should begin with a thorough history, physical examination, and audiometric confirmation. Laboratory tests are primarily used to exclude known etiologies (such as infection, autoimmune disease, or neoplasms) and should be regarded as a complement to, rather than a replacement for, standard audiological assessment and guideline-based etiological evaluation (1). All laboratory biomarkers discussed in this review are intended to provide supplementary information for prognosis assessment or etiological hypothesis, not for routine diagnosis. Currently, there remains a lack of specific laboratory indicators that can reliably reflect disease activity, enable etiological classification, or predict prognosis.Therefore, in-depth exploration of its pathogenesis and the search for effective laboratory diagnostic markers have become research hotspots and challenges in this field. In recent years, researchers have attempted to find potential biomarkers from multiple perspectives. For example, studies have explored the expression levels of circulating microRNAs and found that the combination of miR-183, miR-210, miR-18b, and miR-23a showed high sensitivity and specificity in distinguishing ISSNHL patients from healthy controls (18). Serum levels of prestin, a protein specific to cochlear outer hair cells, are elevated in ISSNHL patients, and its dynamic changes may be related to hearing recovery (19). In addition, hematological indicators reflecting systemic inflammatory status, such as the neutrophil-to-lymphocyte ratio, platelet-to-lymphocyte ratio, and systemic immune-inflammation index, have also been found to be associated with the prognosis of ISSNHL (20, 21). In exploring etiology, some studies have indirectly assessed vascular status by evaluating arterial stiffness (e.g., brachial-ankle pulse wave velocity, cardio-ankle vascular index) and found that these indicators are related to the onset and severity of ISSNHL (22, 23). However, a large national database study showed that although the positivity rate of non-specific autoimmune laboratory markers was slightly higher in ISSNHL patients, the presence of these markers did not predict treatment response or prognosis (24). Notably, although previous narrative overviews have summarized the vascular, inflammatory, and immune-related biomarkers of ISSNHL, few prior narrative collations assemble multimodal biomarkers into a unified analytical framework. The present narrative overview puts forward several distinctive analytical perspectives: (i) integrated collation and comparative discussion of sensitivity and specificity across diverse blood biomarker subgroups (inflammation, coagulation, autoantibodies, metabolism) and the proposal of a multi-marker combined modeling strategy for clinical translation; (ii) the integration of metabolomics and immune indicators within one shared analytical framework, revealing the synergistic role of metabolic–immune crosstalk in the pathogenesis and prognosis of ISSNHL; and (iii) the incorporation of recent advances (2024–2026), including exosomal miRNAs/proteins, complement C3, and AI-assisted personalized treatment, thereby filling the gaps left by reviews published in the last three years. This article provides a narrative review of the current literature on the pathogenesis and laboratory biomarkers of ISSNHL. It is not a systematic review, and no formal search strategy, selection criteria, or quality assessment tools were pre-specified. Instead, we aimed to synthesize key findings from representative studies to offer a conceptual framework for understanding the disease.

## 2 Pathogenesis of ISSNHL

**Figure 1:**
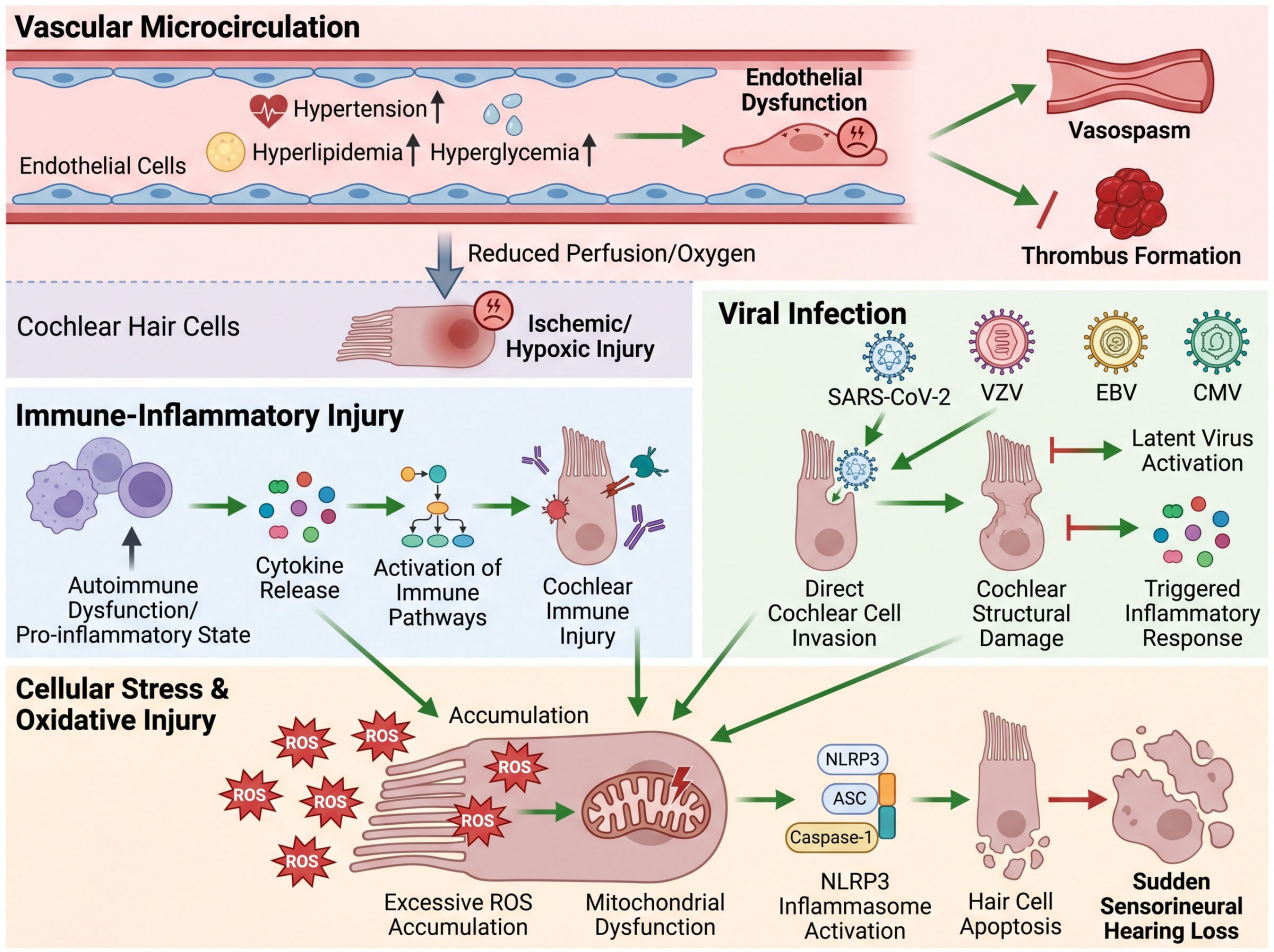
Pathophysiological mechanisms of idiopathic sudden sensorineural hearing loss (ISSNHL). The pathways depicted are simplified conceptual interactions and are not intended to imply a linear or uniform sequence in all patients. The relative contribution and timing of each mechanism remain uncertain and likely vary among individuals. This figure is original and was created by the authors using BioGDP (https://biogdp.com/), a biomedical graphic design software; the software was used solely as a drawing tool, and all scientific content was designed, reviewed, and validated by the authors.

The occurrence of ISSNHL has been hypothesized to involve the synergistic and progressively amplified effects of vascular microcirculatory disturbances, immune-inflammatory damage, viral infections, cellular stress, and oxidative injury. The inner ear is supplied by terminal arteries with no collateral circulation, making it highly susceptible to ischemia, hypoxia, inflammation, and viral attacks. The mechanism diagram is shown in **Figure 1**.

### 2.1 Microcirculatory disorders and vascular factors

Since the inner ear is supplied by end arteries and is extremely sensitive to ischemia and hypoxia, cochlear microcirculatory disorders (cochlear ischemia or cochlear infarction) caused by vascular lesions due to vasospasm, thrombosis, embolism, or hemorheological abnormalities are one of the core pathogenic mechanisms of ISSNHL (5). Basic experiments using a gerbil model of transient cerebral ischemia have confirmed that vascular lesions activate the inducible nitric oxide synthase (iNOS) molecular pathway, leading to abnormal accumulation of nitric oxide (NO). This effect further aggravates vascular endothelial dysfunction and promotes the occurrence of cochlear ischemic injury (25). Intervention experiments in a gerbil model of cochlear ischemia have shown that the antioxidant molecule hydrogen can target and ameliorate endothelial injury, block the ischemic cascade, and effectively reverse cochlear ischemia-induced hearing loss. These animal experiments have confirmed that vascular-origin cochlear ischemia is a key pathogenic mechanism of ISSNHL (26). A large body of basic experimental evidence further confirms that the internal auditory artery functions as a terminal artery lacking collateral circulation, so it is unable to compensate once vascular occlusion occurs. Therefore, any disease that interrupts cochlear perfusion may ultimately lead to reduced oxygen supply to the cochlea and trigger ISSNHL. Experimental evidence shows that the cochlear microcirculation is extremely sensitive to changes in blood flow. In fact, even mild hypoperfusion can lead to immediate loss of function of the organ of Corti; cochlear hair cells, due to their high metabolic activity, are highly susceptible to hypoxic or ischemic injury (27, 28). Vascular endothelial cells lining the inner wall of cochlear vessels play a crucial role in regulating vascular tone and maintaining microcirculatory homeostasis. By regulating hemodynamics, maintaining immune homeostasis, and synthesizing key signaling molecules such as nitric oxide, they provide oxygen and nutrients to tissues, regulate vascular tone and permeability, maintain hemostasis and coagulation, induce angiogenesis, and coordinate inflammatory responses (29) When endothelial cell dysfunction occurs, it can induce endothelial dysfunction, mainly manifested as impaired vasodilation, enhanced oxidative stress response, and activation of a pro-inflammatory phenotype. Recent studies have confirmed a significant correlation between endothelial dysfunction and ISSNHL, suggesting that it may affect the blood perfusion of the cochlea (the auditory organ of the inner ear) by disrupting hemodynamic homeostasis. Such hypoperfusion can lead to a deficiency of oxygen and nutrient substrates for the highly metabolically demanding cochlear hair cells, thereby inducing cell damage and even apoptosis, ultimately exacerbating the vascular endothelial pathological process (2).

Recent studies on lipid metabolism have further enriched the evidence for the vascular etiology. Observational data suggest that serum lipid metabolite profiles—including remnant cholesterol and lysophospholipids—differ significantly between ISSNHL patients and healthy controls (30, 31). These findings provide new clinical support for the vascular hypothesis from the perspective of lipid metabolism. Yukihito et al. (7) showed that dyslipidemia is a key pathological factor inducing endothelial dysfunction, and its damage mechanism presents a multi-step cascade effect: in the process of lipoprotein metabolism, harmful lipoproteins such as s-d-LDL, RLP, and oxLDL generated from LDL and VLDL through the action of enzymes like CETP and LPL can trigger oxidative stress and inflammatory responses, thereby interfering with intracellular signaling pathways in endothelial cells. On the one hand, this inhibits eNOS activity and induces its uncoupling via ROCK, reducing NO bioavailability; on the other hand, it drives excessive ROS production through NADPH oxidase, affecting gene expression through redox signaling disturbances. Ultimately, this series of abnormalities leads to an imbalance in the response of endothelial cells to vasoactive factors, decreased proliferation and migration capacity, increased senescence and apoptosis, and irreversible endothelial injury. These mechanisms elucidate at the molecular level how vascular lesions progressively lead to cochlear ischemia and hair cell damage. Notably, cardiovascular risk factors have been shown to have predictive value for the prognosis of sudden hearing loss. For instance, Ogreden et al. (32) demonstrated that the CHA_2_DS_2_-VASc score has prognostic significance in sudden sensorineural hearing loss, further supporting the critical role of vascular factors in its pathogenesis. Furthermore, Özkan et al. (33) found significantly elevated oxidative stress markers and impaired antioxidant defense in patients with idiopathic sudden sensorineural hearing loss in a case-control study, providing additional clinical evidence for the oxidative stress mechanism.

### 2.2 Immune- and inflammation-mediated injury mechanisms

Immune and inflammatory responses play a significant role in the pathogenesis of ISSNHL. Autoimmune inner ear disease (AIED) is considered an etiological factor in some ISSNHL cases, classified as primary (involving only the inner ear) or secondary (associated with systemic autoimmune diseases [SAID]) (3). A variety of cytokines and inflammatory mediators, including interleukins (ILs), tumor necrosis factors (TNFs), and interferons (IFNs), are widely studied for their involvement in local immune responses and tissue damage in the inner ear (20, 34). Imbalance of helper T cell (Th) subsets (e.g., Th1, Th2, Th17)-related factors may contribute to immune-mediated ISSNHL. Experimental evidence suggests that a systemic pro-inflammatory state may participate in the pathophysiological mechanisms of hearing loss by affecting cochlear hair cells and stria vascularis cells (3, 24). In recent years, the role of the complement system in the immune mechanism of ISSNHL has garnered attention. A 2024 study reported that complement C3 levels on plasma extracellular vesicles (EVs) were closely correlated with the severity and treatment response of severe sudden sensorineural hearing loss (SSNHL; noting that the majority of such patients would be idiopathic, i.e., ISSNHL), suggesting that complement activation may directly damage cochlear hair cells or the stria vascularis by forming membrane attack complexes or promoting inflammatory cascades (35). This finding extends the immune-mediated injury mechanism from the cytokine level to the complement system, offering a new perspective for understanding the immune heterogeneity of ISSNHL.

Importantly, the heterogeneity of immune findings across studies is substantial and reflects several sources: (1) varying case definitions and inclusion criteria (e.g., bilateral vs. unilateral ISSNHL, severity thresholds); (2) timing of blood sampling relative to symptom onset, which critically influences cytokine and inflammatory marker levels; (3) differences in assay platforms and laboratory protocols across institutions; and (4) confounding by concurrent systemic conditions such as autoimmune diseases, infections, or corticosteroid treatment. These factors collectively limit direct comparability across cohorts and underscore the need for standardized protocols in future prospective studies.

### 2.3 Viral infection theory

Viral infection is considered one of the important potential causes of Idiopathic Sudden Sensorineural Hearing Loss (ISSNHL). Various viruses, including SARS-CoV-2, cytomegalovirus (CMV), varicella-zoster virus (VZV), and Epstein-Barr virus (EBV), It is important to distinguish ISSNHL caused by identifiable viral infections (e.g., Ramsay Hunt syndrome, congenital CMV) from truly idiopathic cases, where viral causality remains speculative. These pathogens may directly infect inner ear hair cells, supporting cells, or the stria vascularis, or induce reactivation of latent viruses, leading to acute damage to the structure and function of the inner ear (36, 37).

A prospective case-cohort study by Swaminathan, B. et al. (38)evaluated the hearing characteristics of 50 asymptomatic adults with RT-PCR-confirmed COVID-19 infection and their age- and sex-matched healthy controls using pure-tone audiometry (PTA). It found that sensorineural hearing loss (SNHL) patterns were common in COVID-19 infection. In adults infected with SARS-CoV-2, transient but severe cochlear dysfunction can cause transient high-frequency sensorineural hearing loss. The analysis suggests that COVID-19, like other viral infections, may directly damage inner ear structures, hair cells, and cortical cells, or indirectly affect the inner ear and brainstem through host immune-mediated mechanisms in severe cases (39, 40). But these associations should be interpreted cautiously; causality has not been established, and confounding factors (e.g., stress, delayed presentation) may contribute. A retrospective study by Thielker, J. et al. (41) reviewed 574 hospitalized patients treated for sudden hearing loss, of whom 490 patients (85%) were diagnosed with ISSNHL and 84 patients (15%) with NISSNHL (non-idiopathic sudden sensorineural hearing loss). Among the NISSNHL cases, 49% had hearing loss due to acute otitis media, 37% due to varicella-zoster infection or Lyme disease, 10% due to Meniere’s disease, and 7% due to other causes. There were no differences between ISSNHL and NISSNHL patients in terms of age, sex, side of hearing loss, presence of tinnitus or vertigo, and comorbidities. Multiple case reports have shown that varicella infection can present with sudden deafness as the first symptom, suggesting a direct association between VZV infection and inner ear damage (13, 42).

However, studies detecting viral DNA/RNA or specific antibodies in peripheral blood or inner ear samples of patients to support this hypothesis have yielded controversial positivity rates, and it is difficult to distinguish whether viral infection is a direct cause of ISSNHL or a concomitant phenomenon. The latest molecular biology techniques, such as high-throughput sequencing, are being attempted to identify unknown viral pathogens in inner ear samples or blood, thereby providing more direct evidence for the viral infection theory (43, 44). However, it must be emphasized that most evidence for viral causation in ISSNHL is indirect (case reports, retrospective studies), and definitive proof of direct viral invasion of the cochlea is lacking.

### 2.4 Cellular stress and oxidative damage mechanisms

Cellular stress and oxidative damage are a key convergence point in the pathophysiological process of ISSNHL. The cochlea has a high metabolic rate and is particularly sensitive to oxidative stress damage. Various initiating factors, such as ischemia and viral infection, may ultimately lead to excessive production of reactive oxygen species (ROS) in the cochlea, disrupting the balance of the antioxidant defense system, and thereby inducing hair cell apoptosis (45, 46).It should be noted that much of this mechanistic understanding is derived from experimental cochlear injury models (e.g., noise-, drug-induced) and age-related hearing loss studies; direct evidence specifically from ISSNHL patients remains limited. Laboratory research has provided evidence for this, showing that the balance between oxidative stress and antioxidants is disrupted in ISSNHL patients.

A study by Çetin, Y. S. et al. (47) compared serum redox indicators between the recovery and non-recovery groups of ISSNHL patients. They found significant differences between the two groups in indicators such as total oxidant status, disulfide, disulfide/native thiol percentage, disulfide/total thiol percentage, and native thiol/total thiol ratio, indicating that elevated oxidative stress levels and thiol-disulfide homeostasis imbalance are common in ISSNHL patients. These findings support the hypothesis that vascular pathology is the main cause of ISSNHL and suggest that other etiological factors may ultimately act by inducing vascular pathology and oxidative damage. Mitochondrial dysfunction, as a key link in cellular energy crisis and apoptosis, plays a crucial role in the pathogenesis of sudden deafness. ROS-induced mitochondrial dysfunction, along with NLRP3 inflammasome activation, jointly participates in cochlear damage (48). Neuroprotective treatment strategies targeting oxidative damage are being explored. For example, intratympanic injection (ITE) of the free radical scavenger edaravone has shown potential in clinical trials for treating severe ISSNHL. Compared with historical controls receiving intratympanic steroid injection (ITS), the ITE group showed more significant hearing improvement in the low-frequency range and a greater average degree of hearing improvement, providing a new approach to improving prognosis by targeting oxidative damage (49).This finding is one of the few direct interventional evidences in ISSNHL patients supporting the oxidative stress hypothesis. Beyond direct oxidative stress, metabolic homeostasis imbalance may also participate in the pathogenesis of ISSNHL by inducing cellular stress. A 2026 Mendelian randomization study utilizing genetic instrumental variables for cerebrospinal fluid (CSF) metabolites and ISSNHL risk identified several metabolites (e.g., sphingomyelins, glutamine) that causally influence ISSNHL risk, suggesting that metabolic abnormalities in the inner ear microenvironment can independently induce cellular damage (50). From a causal inference perspective, this study supports the potential role of cellular metabolic stress in the pathogenesis of ISSNHL. However, this evidence is based on genetic instrumental variable analysis and remains indirect; direct metabolomic profiling in ISSNHL patient cohorts is needed for validation. To clarify the mainstream pathogenic hypotheses of ISSNHL, we summarize the underlying mechanisms, evidence and limitations in **Table 1**.

**Table 1:** Summary of pathogenic mechanisms of ISSNHL.

Pathogenic mechanism	Core mechanism	Type of evidence	Limitations	Readiness	References
Vascular and endothelial injury	Vasospasm, thrombosis, and endothelial dysfunction lead to cochlear ischemia and hypoxia; dyslipidemia exacerbates vascular damage	Animal experiments, case-control studies, clinical cohorts	Most findings are associative; easily confounded by hypertension, diabetes, and other comorbidities	High	(7, 25, 26, 32, 33)
Immune and inflammatory injury	Cytokine release and autoimmune responses damage cochlear structures; AIED accounts for ~6.2% of SSNHL cases (majority idiopathic)	Case-control studies, large-scale database analysis	Non-specific immune markers cannot predict treatment response or long-term prognosis; substantial inter-study heterogeneity due to varying definitions and assay timing	Moderate	(3, 24, 34, 35)
Viral infection hypothesis	Viruses invade inner ear tissues or reactivate latent viruses and cause structural damage	Retrospective studies, case reports	Only epidemiological correlations observed; direct evidence of viral invasion into the human cochlea is lacking; causality unestablished	Low	(13, 38, 41)
Cellular stress and oxidative damage	Excessive ROS, mitochondrial dysfunction, and thiol-disulfide imbalance trigger cochlear hair cell apoptosis	Animal experiments, case-control studies, Mendelian randomization	Most mechanistic data are derived from animal models; direct evidence in ISSNHL patients remains limited	Moderate	(45, 47, 49, 50)

## 3 Exploration of laboratory diagnostic indicators based on hematology

### 3.1 Coagulation and fibrinolysis system indicators

Based on the vascular hypothesis of ISSNHL, coagulation and fibrinolysis markers can serve as risk factors (e.g., hyperfibrinogenemia, elevated D-dimer), etiological markers (reflecting thrombosis), or prognostic indicators (predicting recovery). However, they are not diagnostic biomarkers for ISSNHL, as they lack specificity and are commonly influenced by comorbidities. The following sections outline their proposed clinical roles in risk stratification and prognosis assessment.

D-dimer, as a specific product of fibrin degradation, directly reflects the presence of a hypercoagulable state or secondary hyperfibrinolysis in the body. Multiple studies have reported (51, 52)that serum APTT, PT, FIB, and D-dimer levels can serve as prognostic predictors of ISSNHL severity, especially when used in combination (AUC = 0.91, sensitivity = 86.76%, specificity = 82.61% in one single-center cohort). Patients with higher degrees of hearing loss (>91 dB) had significantly lower APTT and PT values and higher serum FIB and D-dimer levels compared with those with milder loss. These findings should be interpreted with caution given the single-center design and lack of external validation.

Fibrinogen, as a key protein in the coagulation process, increases whole blood and plasma viscosity when its level is elevated, potentially exacerbating blood stasis and disorders in the inner ear microcirculation. Therefore, the fibrinogen level is a common indicator for evaluating the hemorheological status and predicting the prognosis of ISSNHL patients. In addition, parameters reflecting platelet activation status and function, such as platelet count, mean platelet volume (MPV), and platelet distribution width (PDW), are also associated with the occurrence and development of ISSNHL. Changes in these parameters suggest a potential risk of vascular endothelial damage and thrombosis. A case-control study showed that the proportion of ISSNHL patients with elevated MPV levels was significantly higher than that in healthy controls, further supporting the important role of vascular occlusion in the etiopathogenesis of ISSNHL (53).In addition, clinical studies have found that blood glucose, glycated hemoglobin (HbA1c), lipoprotein(a), and coagulation factor VIII levels are significantly elevated in ISSNHL patients (P < 0.05), and that blood glucose, HbA1c, uric acid, factor VIII, and homocysteine are significantly associated with disease severity (54). elevated fibrinogen increases blood viscosity and can cause vascular thrombosis, increasing the likelihood of ISSNHL onset (55, 56);elevated mean platelet volume (MPV) and neutrophil-to-lymphocyte ratio (NLR) suggest the involvement of vascular pathology (57);and increased brachial-ankle pulse wave velocity (baPWV) is associated with poorer hearing prognosis (22). Clinically, obesity and cardiovascular and metabolic diseases such as hypertension, diabetes mellitus (DM), and hyperlipidemia can reduce vascular elasticity and induce atherosclerosis, thereby causing microvascular lesions. They may also cause changes in the blood vessels and capillaries supplying the ear, disrupting the blood circulation of the inner ear, subsequently damaging hair cells, and ultimately leading to cochlear microcirculatory disorders (58).

Taken together, these coagulation and fibrinolysis indicators provide laboratory evidence supporting the vascular etiology hypothesis of ISSNHL and may facilitate risk stratification and prognosis assessment. Nonetheless, their nonspecific nature and susceptibility to confounding by metabolic comorbidities, infections, and concurrent medications limit their standalone diagnostic utility.

### 3.2 Inflammatory response markers

Inflammatory markers such as NLR, PLR, and SII are primarily prognostic indicators associated with hearing recovery, rather than diagnostic markers. They may also serve as surrogate markers of disease activity (etiological role) in the context of systemic inflammation. Their clinical utility lies in risk stratification, not in establishing the diagnosis of ISSNHL.

Traditional non−specific inflammatory markers: C−reactive protein (CRP) and erythrocyte sedimentation rate (ESR) may be elevated in the acute phase of ISSNHL, but their sensitivity and specificity are limited; their clinical value lies mainly in ruling out other systemic inflammatory or autoimmune diseases (59). Novel inflammatory markers: The neutrophil−to−lymphocyte ratio (NLR), as an easily obtained systemic inflammatory marker, has been widely studied. Elevated NLR is significantly associated with ISSNHL occurrence, severity, and treatment response. Studies show that the mean NLR of ISSNHL patients is significantly higher than that of healthy controls, and an NLR ≥2.01 is a risk factor for ISSNHL (60). Regarding prognosis, non−recovery groups have significantly higher NLR values than recovery groups (20). Furthermore, NLR is linked to the vascular etiology hypothesis: in the subgroup of ISSNHL patients with abnormally elevated NLR, basilar artery dilatation is positively correlated with initial hearing loss severity, suggesting NLR may serve as a marker of vascular−origin ISSNHL (61). Other markers such as the systemic immune−inflammation index (SII) and C−reactive protein−to−albumin ratio (CAR) are significantly elevated in non−recovery groups, indicating that a systemic inflammatory state may be associated with poorer hearing recovery (20, 34).

Regarding sources of inter-study heterogeneity for inflammatory markers: studies differ in their definition of ISSNHL severity (e.g., thresholds of 30 vs. 40 vs. 60 dB), in blood sampling timing relative to symptom onset (ranging from hours to weeks), in assay platforms (automated analyzers vs. manual methods), and in adjustment for confounders such as corticosteroid pre-treatment, concurrent infections, and metabolic comorbidities.

Newer, more specific inflammatory markers are continuously being identified. Soluble CD163 (sCD163), a soluble marker of macrophage activation, was shown in a 2026 study to be significantly associated with disease severity and poor prognosis in SSNHL (original study used SSNHL criteria, with the majority of patients being idiopathic) (62). Furthermore, transferrin, as an acute-phase reactant, demonstrated potential for predicting hearing recovery in SSNHL in a 2025 prognostic study, possibly through mechanisms involving the interplay between iron metabolism and oxidative-inflammation (63). These emerging markers require replication in larger, independent cohorts before clinical adoption.

Autoimmune−related markers: Clinical studies show that the positivity rate of autoimmune laboratory markers in ISSNHL patients (23%) is marginally significantly higher than in non−ISSNHL patients (21%), but the difference disappears after excluding highly non−specific markers (e.g., rheumatoid factor, antinuclear antibody) (24). Meanwhile, a study of 694 SSNHL patients found an AIED prevalence of 6.2%, with primary AIED accounting for 83.7% and secondary AIED 16.3% (co−existing with Sjögren’s syndrome and systemic lupus erythematosus) (3). These data suggest that targeted autoantibody testing may be more clinically meaningful for patients with suspected autoimmune symptoms.Taken together, these inflammatory and autoimmune markers provide laboratory evidence for etiological classification of ISSNHL and development of individualized treatment strategies.

### 3.3 Immunological and autoantibody-related diagnostic indicators

Autoantibodies can be classified based on their clinical role: etiological markers (e.g., anti-cochlin, anti-prestin in selected cases), risk factors (e.g., non-specific autoantibodies in the context of systemic autoimmune disease), and prognostic markers (limited utility). However, due to low specificity and poor reproducibility, none of these autoantibodies are currently recommended as diagnostic biomarkers for routine clinical use. Their role is primarily to aid etiological classification in patients with clinical suspicion of autoimmune inner ear disease. Although most cases are classified as “idiopathic,” increasing evidence suggests that the onset in some patients is closely related to autoimmune responses, providing direction for the exploration of laboratory diagnostic indicators. Detecting specific autoantibodies can not only help identify patient subgroups with a potential autoimmune background but also provide clues for understanding the pathophysiological mechanisms of inner ear damage, thereby guiding more targeted treatment strategies, such as the application of immunosuppressive therapy (64).

#### 3.3.1 Non-organ-specific autoantibodies

In patients with Idiopathic Sudden Sensorineural Hearing Loss, detecting non-organ-specific autoantibodies, such as antinuclear antibodies and anticardiolipin antibodies, is valuable for screening patients with a background of systemic autoimmune diseases. A national study based on a large US medical database explored this (24, 51), comparing the positivity rates of autoimmune laboratory markers between ISSNHL patients receiving systemic steroid treatment and a control population. The results showed that the proportion of ISSNHL patients with at least one positive autoimmune marker (23.0%) was slightly higher than that in the non-ISSNHL population (21.4%), but this difference lost statistical significance after excluding non-specific autoimmune markers. More importantly, the study found no significant differences between autoantibody-positive and -negative patients in the rates of requiring salvage intratympanic steroid injection, hearing aid evaluation, or cochlear implantation, indicating that the presence of these non-specific autoantibodies cannot predict treatment response or overall prognosis. Therefore, although detecting systemic autoantibodies can help identify patients who may have concurrent systemic autoimmune diseases, extensive autoimmune screening for ISSNHL patients without other suspicious symptoms may not alter their clinical management or prognostic judgment. It is noteworthy that beyond classical autoantibodies, components of the complement system can also serve as non-specific markers of immune activation. Recent research indicates that the level of complement C3 on plasma extracellular vesicles can serve as a serum marker for assessing disease severity and treatment response in SSNHL, and its clinical diagnostic value warrants further validation (35). This marker, together with the aforementioned autoantibodies, enriches the immunological diagnostic indicator spectrum for ISSNHL.

Anti-endothelial cell antibodies are another class of non-organ-specific autoantibodies that may be involved in the pathogenesis of ISSNHL (65). Their mechanism may be related to damaging the vascular endothelium of the inner ear. Vascular damage is one of the main etiological hypotheses for ISSNHL, and anti-endothelial cell antibodies can affect the inner ear microcirculation by triggering vascular inflammatory reactions, leading to ischemic damage. A study by Gündoğan, F. et al. (66) measured plasma levels of endothelial cell-specific molecule-1 (ESM-1) and pentraxin-3 (PTX-3) in 108 subjects, including 51 ISSNHL patients and 57 healthy controls. They also compared the differences in these indicators between ISSNHL patients before treatment and after 3 months of treatment, as well as between treatment responders and non-responders. The results showed that plasma ESM-1 levels in ISSNHL patients were significantly higher than those in the control group, while PTX-3 levels showed no difference between groups. Furthermore, there were no statistically significant differences in either indicator before and after treatment in all patients. These results suggest that endothelial dysfunction mediated by elevated ESM-1 levels may be involved in the pathogenesis of ISSNHL, and its onset may be related to vascular damage.

#### 3.3.2 Inner ear-specific or related autoantibodies

Autoantibodies against inner ear-specific or related antigens provide more direct evidence for exploring the autoimmune etiology of Idiopathic Sudden Sensorineural Hearing Loss (10, 67). Cochlin, an extracellular matrix protein, is homologous to a key serine protease C involved in the antibacterial response in horseshoe crabs. Mutations in the COCH gene are the pathogenic cause of human DFNA9 syndrome, characterized by neurodegenerative changes in the ear, leading to hearing loss and vestibular dysfunction (68). Cochlin is highly expressed in the inner ear, especially in the spiral ligament and basilar membrane of the cochlea (69–71). Anti-cochlin antibodies are specific antibodies targeting the cochlear protein Cochlin encoded by the COCH gene. Boulassel, MR et al. found that the 58 kDa target protein of antibodies in the serum of patients with autoimmune inner ear disease is the COCH5B2 protein, a molecule highly specifically expressed in the cochlea and vestibule, which has attracted considerable attention (72, 73). Although current research literature on this specific autoantibody is limited, as a new diagnostic target, it holds promise for clinical diagnostic application research (69–71). Prestin (gene symbol: SLC26A5) is a homologous protein of the SLC26 anion transporter family. As a motor protein specifically and highly expressed in the lateral membrane of outer hair cells (OHCs) of the mammalian cochlea, its core function is to mediate voltage-dependent cell body length changes (i.e., electromotility), which is the molecular basis of the cochlear amplifier. It directly determines the sensitivity (can enhance by 40–60 dB) and frequency selectivity of mammalian hearing, playing a central role in cochlear sensitivity and tuning. Anti-prestin antibodies are autoantibodies that damage the inner ear through cross-reactivity of circulating antibodies with inner ear antigens or activated T cells (19, 74). Anti-β-actin antibodies and anti-heat shock protein 70 antibodies are potential markers for ISSNHL. However, the specificity of these antibodies remains highly controversial (75). For example, heat shock proteins are widely expressed under stress conditions, and their antibodies can appear in various infectious or inflammatory states and are not unique to inner ear diseases. Studies in the references also indirectly reflect the challenge of finding specific markers. A study on the prevalence of autoimmune inner ear disease in sudden sensorineural hearing loss found that among 694 patients, only 6.2% were diagnosed with AIED, most of whom (83.7%) had primary disease (affecting only the inner ear) (75). This suggests that cases truly mediated by specific autoantibodies may only constitute a small proportion, and diagnosis relies on multifaceted clinical evaluation rather than a single antibody test. Therefore, for anti-β-actin antibodies, anti-heat shock protein 70 antibodies, anti-prestin antibodies, anti-cochlin antibodies, etc., to become reliable diagnostic tools, more strictly standardized detection methods (to reduce false positive and false negative rates) and validation in larger, multi-center studies are needed to clarify their precise relationship with disease type, activity, and prognosis.

It should be emphasized that although autoimmune injury is one of the proposed mechanisms, only a minority of ISSNHL cases are clearly autoimmune (e.g., bilateral/recurrent disease, steroid dependency, or concurrent systemic autoimmune disorders). Therefore, routine broad autoimmune screening is not recommended for all ISSNHL patients. Such testing should be reserved for selected patients with clinical suspicion. The clinical specificity and reproducibility of many autoantibodies (e.g., anti-HSP70, anti-prestin, anti-cochlin) remain uncertain, limiting their routine clinical utility. Conventional blood-derived biomarkers are summarized below, including specimen types, functional roles, research data, evidence and clinical limitations **Table 2**.

**Table 2:** Summary of hematological, coagulation, inflammatory and autoimmune biomarkers.

Biomarker category	Specific indicators	Specimen type	Functional role	Key research data	Type of evidence	Limitations	Readiness	References
Coagulation and fibrinolysis markers	APTT, PT, FIB, D-dimer	Peripheral venous blood	Etiological & prognostic	Combined detection: AUC = 0.91, Sens=86.76%, Spec=82.61%; abnormal in patients with hearing loss >91 dB	Clinical cohort	Low disease specificity; affected by thrombosis, infection, medications; single-center only	High	(51, 52)
Platelet-related markers	MPV, PDW	Peripheral venous blood	Etiological	MPV levels significantly elevated in ISSNHL patients	Case-control	No unified clinical cut-off value	Moderate	(53)
Multiplex inflammatory markers	NLR, PLR, SII, CAR	Peripheral venous blood	Prognostic	NLR ≥2.01 is a risk factor; higher NLR indicates poor hearing recovery; meta-analyses confirm association but report high heterogeneity	Meta-analysis, clinical cohort	Universal inflammatory indicators without disease specificity; inter-study heterogeneity from varying definitions, sampling timing, and confounders	High	(20, 60, 76, 77)
Routine inflammatory markers	CRP, ESR	Peripheral venous blood	Auxiliary differential	Elevated in acute phase of ISSNHL	Retrospective cohort	Low sensitivity and specificity	Moderate	(59)
Non-specific autoantibodies	ANA, anticardiolipin antibody	Peripheral venous blood	Etiological screening	Positive rate: 23% (ISSNHL) vs 21% (control)	Large-scale database	Unable to predict treatment response or prognosis; not recommended for routine screening	Low	(24)
Endothelial markers	ESM-1, PTX-3	Peripheral venous blood	Etiological	ESM-1 significantly increased in patients (n=108)	Case-control	No obvious dynamic changes after treatment; small sample size	Very low	(66)
Inner ear-specific autoantibodies	Anti-cochlin, Anti-prestin, Anti-HSP70	Peripheral venous blood	Etiological (AIED subtype)	Detected in selected AIED patients	Small-sample study	Non-standard detection methods; high false-positive rate; low reproducibility	Very low	(74, 75, 78)

## 4 Emerging molecular and metabolomic diagnostic indicators: a perspective

### 4.1 Circulating nucleic acids and epigenetic markers

Emerging molecular markers such as circulating miRNAs and exosomal proteins have shown promise as both diagnostic biomarkers (distinguishing ISSNHL from controls) and prognostic biomarkers (predicting recovery). However, these markers remain in the exploratory research phase and have not been validated for routine clinical use as diagnostic tools. Their potential role in etiological classification (e.g., distinguishing ischemic from inflammatory subtypes) warrants further investigation. Circulating MicroRNAs, as a class of stable molecular markers, exhibit significantly altered expression profiles in the plasma of ISSNHL patients, demonstrating potential as novel diagnostic and prognostic prediction tools. A 2026 study established a model for predicting the prognosis of severe SSNHL by analyzing circulating exosomal miRNA profiles, suggesting specific miRNA combinations could serve as tools for early prognostic assessment (79). Concurrently, a 2025 study identified serum exosomal proteins RPS2, RPL19, ACO2, and APOE as diagnostic biomarkers for SSNHL, whose expression changes may reflect inner ear neuronal damage or oxidative stress (80).These achievements mark the transition of ISSNHL liquid biopsy from traditional serum markers to the era of exosome omics.

A study by Ha, S. M., et al. (18) using real-time quantitative polymerase chain reaction found significant differences in the expression levels of various circulating MicroRNAs, including miR-183, miR-210, miR-18b, and miR-23a, between ISSNHL patients and healthy controls. These differentially expressed MicroRNAs are closely associated with pathophysiological processes such as Angiogenesis, inflammation, and Apoptosis. Their expression levels are not only correlated with patients’ hearing ability but also with treatment outcomes. By jointly evaluating the cut-off values of these specific MicroRNAs, the study achieved a sensitivity of up to 80.95% and specificity of 87.50%, providing a strong molecular basis for the non-invasive diagnosis of ISSNHL. Furthermore, cell-free DNA levels, as a potential indicator reflecting the degree of tissue damage, warrant further exploration regarding their relationship with the severity of hearing loss in ISSNHL. Although direct studies on the relationship between cell-free DNA and ISSNHL are limited in current literature, based on the principle that it may be released into the bloodstream after inner ear ischemia or inflammatory injury, it holds promise as a complementary marker for assessing the severity of cochlear damage. Meanwhile, epigenetic modifications such as DNA methylation are thought to be involved in the pathogenesis of ISSNHL. Epigenetic regulation influences gene expression without altering the DNA sequence and plays a crucial role in responding to stimuli such as environmental stress, inflammation, and ischemia. Exploring the methylation status of inner ear-specific genes or genes related to vascular and immune pathways can help elucidate the molecular pathogenesis of ISSNHL and point towards new directions for developing early warning or classification diagnostic tools based on epigenetic markers.

### 4.2 Metabolomic and lipidomic characteristics

Metabolomic and lipidomic alterations (e.g., elevated bile acids, lysophospholipids) are best classified as risk factors or disease activity markers reflecting oxidative stress and metabolic dysregulation. They are not yet established as diagnostic biomarkers due to limited evidence from small-scale exploratory studies. Their future role may be as prognostic indicators or subtype classifiers once validated.

Endolymphatic metabolic homeostasis is essential for cochlear function. Metabolic disturbances in the endolymph, particularly elevated glutamate and reduced adenosine triphosphate (ATP), have been directly linked to hearing loss: glutamate accumulation induces excitotoxicity in hair cells, while ATP depletion disrupts the endocochlear potential, collectively contributing to inner ear damage. Beyond local metabolic abnormalities in the inner ear, peripheral metabolic disturbances also participate in the pathogenesis of ISSNHL. Untargeted metabolomic analyses have revealed significant alterations in the serum metabolomic profiles of ISSNHL patients. Levels of bile acids, phospholipids, and sphingomyelins are significantly associated with the degree of hearing loss, and upregulation of various lipid metabolites further confirms the role of lipid dysmetabolism in disease progression (81). Moreover, studies linking gut microbiota and fecal metabolomics have drawn similar conclusions: ISSNHL patients commonly exhibit gut dysbiosis, and differential fecal metabolites are primarily enriched in amino acid biosynthesis pathways, providing additional evidence for the involvement of systemic metabolic disturbances (82).

Lipidomics focuses on the pathological alterations of lipid molecules. In the context of ISSNHL, elevated oxidized lipids and lysophospholipids are considered important markers of oxidative damage. Oxidative stress is a well-recognized pathogenic mechanism in ISSNHL: excessive accumulation of reactive oxygen species (ROS) triggers lipid peroxidation in inner ear hair cells, compromising cell membrane structure and function (83). Lysophospholipids, generated from membrane phospholipids by phospholipase A_2_, serve both as indicators of phospholipid degradation and as signaling molecules mediating inflammatory responses.It should be emphasized that direct metabolomic and lipidomic studies specifically targeting ISSNHL remain relatively limited at this stage, with most findings derived from small-scale exploratory analyses. Therefore, the clinical translational value of these metabolic markers, particularly their reliability as diagnostic or prognostic tools, still requires validation through large-scale, multicenter prospective studies. Novel molecular and metabolomic biomarkers are emerging research hotspots. Their research status, data and limitations are presented in **Table 3**.

**Table 3:** Summary of molecular, epigenetic, metabolomic and lipidomic biomarkers.

Biomarker category	Specific indicators	Specimen type	Functional role	Key research data	Type of evidence	Limitations	Readiness	References
Circulating microRNAs	miR-183, miR-210, miR-18b, miR-23a	Peripheral plasma	Diagnostic & prognostic (exploratory)	Combined detection: Sens=80.95%, Spec=87.50% in single-center cohort	Clinical cohort	No unified detection protocols; no external validation; small sample sizes	Low	(18)
Exosome-derived markers	Exosomal miRNA, RPS2, APOE, RPL19, ACO2	Peripheral plasma	Diagnostic & prognostic (exploratory)	Significant differential expression observed in patient groups	Exploratory cohort	Very small sample size; lack of external validation; standardization challenges	Very low	(79, 80)
Serum metabolites	Bile acids, phospholipids, sphingomyelins	Peripheral serum	Etiological & severity markers	Metabolite levels correlate with hearing loss severity	Exploratory metabolomics	Small-sample exploratory analyses only; no multi-center validation	Low	(81)
Fecal metabolites	Amino acid profiles	Feces	Systemic metabolic markers	Gut dysbiosis detected in ISSNHL patients	Fecal metabolomics	Only indirect correlation with the disease; significant confounding possible	Very low	(82)
Oxidative lipid markers	Oxidized lipids, lysophospholipids, 8-iso-PGF2α	Peripheral serum	Oxidative damage markers	Reflect the level of lipid peroxidation	Lipidomics	Non-specific for ISSNHL; found in many inflammatory conditions	Low	(83)

## 5 Clinical application challenges and prospects

### 5.1 Limitations in the clinical validation of existing biomarkers

Although significant progress has been made in the study of hematological biomarkers for ISSNHL, their clinical translation still faces substantial challenges. First, most studies are small-sample, single-center designs lacking large-scale, multicenter prospective validation, resulting in insufficient reliability and reproducibility. Systematic reviews have indicated that while inflammatory markers such as NLR and PLR are significantly elevated in ISSNHL patients, heterogeneity among studies is high and the quality of evidence is low (76, 77). Such cross-study inconsistency mainly originates from non-uniform blood sampling windows relative to symptom onset, inconsistent inclusion criteria for hearing loss severity and accompanying comorbidities, as well as divergent detection platforms and self-defined threshold values across different research teams. These methodological differences hinder direct horizontal comparison of biomarker efficacy and readily produce conflicting statistical outcomes across independent cohorts. Establishing unified research specifications is therefore essential to reconcile inconsistent findings in subsequent prospective investigations.Second, individual biomarkers rarely achieve both high sensitivity and specificity, with diagnostic performance often at a moderate level, rendering them insufficient for standalone diagnosis (84, 85). Consequently, multi-marker combination modeling has become an important strategy; for example, a prognostic nomogram incorporating age, onset time, vertigo, NLR, MHR, and CAR achieved an AUC of 0.945, significantly outperforming any single marker (86). Furthermore, individual variability, comorbidities (e.g., metabolic syndrome, diabetes), and genetic factors (e.g., suPAR levels influenced by multiple loci with a heritability of 60%) all interfere with the establishment of unified baseline cutoffs (8, 87–89). A critical caveat is that most of the hematological markers discussed (e.g., NLR, PLR, SII, FIB, D-dimer, MPV) are nonspecific systemic inflammatory or coagulation markers. Their levels can be significantly influenced by comorbidities such as metabolic syndrome, diabetes, infection, medication use (e.g., steroids, anticoagulants), age, and smoking status. Therefore, their diagnostic specificity for ISSNHL is inherently limited, and they cannot reliably distinguish ISSNHL from other conditions causing similar systemic changes. Future studies should control for these confounders and evaluate the incremental diagnostic value of these markers beyond clinical judgment.Future efforts should focus on large-scale, multicenter prospective cohorts, standardized detection methods, and stratified reference intervals to facilitate the translation of these biomarkers into clinical practice. To facilitate cross-comparison, we summarize representative high-quality clinical studies in this field **Table 4**.

**Table 4:** Characteristics of high-quality clinical studies on ISSNHL prognosis and treatment.

Study type	Year	Subjects & sample size	Detected indicators	Key statistical results	Main conclusions	Limitations	References
Large-scale database study	2025	ISSNHL vs non-ISSNHL (large national population)	Non-specific autoantibodies	Slight difference in positive rates between two groups; not statistically significant after excluding non-specific markers	Autoantibodies cannot predict prognosis; routine broad screening not justified	Retrospective design; inconsistent inclusion criteria	(24)
Meta-analysis	2018	Combined data from multiple cohorts	NLR	NLR ≥2.01 significantly increases disease risk (P<0.05)	NLR is an independent risk factor but with high inter-study heterogeneity	High heterogeneity; varying definitions and confounders across included studies	(60)
Case-control study	2026	ISSNHL patients vs matched controls	Oxidative stress markers (thiol-disulfide balance)	Increased oxidative stress and impaired antioxidant capacity (P<0.05)	Oxidative imbalance participates in ISSNHL pathogenesis	Single-center; cannot establish causality	(33)
Prospective cohort study	2024	Patients with profound SSNHL (majority idiopathic)	Plasma EV complement C3	Significantly correlated with disease severity and treatment response (P<0.05)	Complement C3 may assess disease activity and treatment efficacy	Relatively small sample size; requires external validation	(35)
Prognostic cohort study	2023	ISSNHL patients	Coagulation indices (APTT, PT, FIB, D-dimer)	Combined model: AUC = 0.91	Coagulation indices have exploratory predictive value	Single-center data without external validation	(52)

### 5.2 Prospects for multi-omics integration and artificial intelligence

Advances in high-throughput sequencing and bioinformatics have made multi-omics integration a powerful tool for deciphering the complex mechanisms of SSNHL. Integrating genomics (e.g., GWAS-revealed susceptibility loci such as FHIT and TRMT1L (8, 87, 88)), proteomics (e.g., GJB2 protein (90)), and metabolomics data can systematically map the complete pathway from genetic predisposition to disease phenotype, enabling the identification of more reliable biomarker panels. Machine learning algorithms (e.g., random forest, logistic regression) are effective in handling high-dimensional, nonlinear data. For instance, a prognostic nomogram based on age, vertigo, fibrinogen (FIB), monocyte-to-lymphocyte ratio (MLR), and CRP/albumin ratio demonstrated a C-index of 0.78 (91), while PLR was identified as an independent risk factor for the prognosis of combined therapy (6)—both confirm the value of multi-marker integration.

AI-assisted personalized diagnosis and treatment is becoming a reality: a 2025 study integrated clinical features with gene polymorphisms (immune- and drug metabolism-related genes) to develop an AI-based prognostic model and individualized treatment plans (92). In the future, longitudinal data modeling based on dynamic monitoring (e.g., time-series analysis of multi-omics data before, during, and after treatment) may enable early identification of poor responders or individuals at high risk of relapse (93). Combined with wearable devices and real-time monitoring, this approach could facilitate the transition from a “one-size-fits-all” treatment paradigm to precision medicine.

Although the application of these techniques in otology is still in its infancy, their enormous potential has already been demonstrated in complex diseases such as cancer (94), providing a theoretical foundation for future exploration. Nevertheless, to translate these promising tools into clinical practice, rigorous validation following a structured roadmap is essential. Specifically, future studies should: (1) adopt prospective, multicenter cohort designs with uniform inclusion/exclusion criteria to assess the reproducibility of existing findings (2); standardize blood sampling timing (e.g., within 24 hours of onset, pre-treatment, and at specific post-treatment intervals) to minimize pre-analytical variability (3); systematically adjust for key confounders including cardiovascular diseases, metabolic syndrome, infections, and medication use (e.g., corticosteroids, anticoagulants) (4); perform external validation in independent cohorts from different centers (5); employ decision-curve analysis to quantify the clinical net benefit of incorporating biomarkers across a range of threshold probabilities; and (6) directly compare the discrimination and calibration of combined biomarker models against established clinical prognostic models (e.g., based on pure-tone thresholds, age, vertigo, etc.) to demonstrate incremental value.

### 5.3 Limitations of this review

Despite this narrative collation of hematological biomarkers in ISSNHL, several methodological limitations should be acknowledged. First, formal structured literature retrieval following standardized frameworks such as the PRISMA guidelines was not performed, which may introduce selection bias and result in the omission of relevant non-English or gray literature. Second, substantial heterogeneity exists across the included studies: definitions of ISSNHL (e.g., hearing loss frequency and threshold criteria) vary considerably; blood sampling timing ranges from hours to weeks after onset; and assay methods differ across laboratories and commercial kits, limiting the comparability and generalizability of findings. Third, treatment regimens are highly heterogeneous—patients received corticosteroids, vasoactive agents, antiviral therapy, or combination regimens, which may confound biomarker levels and prognostic associations. Fourth, confounding by comorbidities (e.g., cardiovascular disease, metabolic syndrome, infections, medication use) was not uniformly adjusted for in most primary studies, reducing the specificity of reported biomarkers for ISSNHL. Fifth, clinically validated biomarker thresholds remain lacking—the majority of cutoff values were derived from single cohorts without external validation in independent populations, precluding their direct application in routine practice. These limitations highlight the need for future studies to adopt standardized protocols, rigorous reporting of potential confounders, and multi-center validation.

## 6 Conclusion

The pathogenesis of idiopathic sudden sensorineural hearing loss (ISSNHL) arises from the overlapping, interactive effects of multiple pathological cascades, including microvascular dysfunction, immune inflammatory activation, viral involvement and cellular oxidative injury. Such heterogeneous multifactorial etiology means no single laboratory marker can fully reflect the varied clinical manifestations of affected patients. Routine hematological indices such as coagulation profiles, inflammatory ratios and circulating autoantibodies may supply limited auxiliary clues for preliminary clinical differentiation, yet their inconsistent performance across patient groups prevents reliable standalone screening or subtyping.

Circulating nucleic acids, exosomal markers and metabolomic signatures show encouraging differentiating signals in small exploratory cohorts; however, these promising preliminary observations are restricted by insufficient multi-center external validation and uniform testing cut-offs, and cannot yet be treated as mature clinical tools.All biomarker categories discussed in this narrative overview remain purely investigational at present. Major translational barriers persist across existing research: most analyses rely on small, single-center samples, while inconsistent inclusion criteria, detection platforms and measurement protocols create substantial inter-study heterogeneity and conflicting outcomes.

For this reason, neither individual indicators nor preliminary multi-marker panels can be recommended for routine diagnostic workup or treatment decision-making. Integrated multi-omics and artificial intelligence analytical pipelines are emerging research avenues; such combined models may offer tentative auxiliary reference for patient stratification only after large prospective validation studies are completed. At this stage, these multi-omics tools cannot deliver definitive diagnostic judgment or standardized individualized therapy guidance.

Overall, biomarker research for ISSNHL is still in the exploratory phase. Standardized testing protocols, cross-population validation and unified threshold values are essential prerequisites before these indicators can be applied to routine clinical care.
